# Supplementary material for: Targeting Feedforward Loops Formed by Nuclear Receptor RORγ and Kinase PBK in mCRPC with Hyperactive AR Signaling
Source: Cancers (Basel). 2021 Apr 1;13(7):1672. doi: 10.3390/cancers13071672 (PMC8036795; doi:10.3390/cancers13071672)
Supplement: Supplementary file 1 [file cancers-13-01672-s001.pdf]

cancers

Supplementary Materials: Targeting Feedforward Loops Formed by Nuclear Receptor RORγ and Kinase PBK in mCRPC with Hyperactivating AR Signaling

Xiong Zhang, Zenghong Huang, Junjian Wang, Zhao Ma, Joy Yang, Eva Corey, Christopher P Evans, Ai-Ming Yu, and Hong-Wu Chen

Citation: Zhang, X.; Huang, Z.; Wang, J.; Ma, Z.; Yang, J.; Corey, E.; Evans, C.P.; Yu, A.-M.; Chen, H.-W. Targeting Feedforward Loops Formed by Nuclear Receptor RORγ and Kinase PBK in mCRPC with Hyperactive AR Signaling. *Cancers* **2021**, *13*, 1672. https://doi.org/10.3390/cancers13071672

Academic Editor: Craig N. Robson

Received: 10 February 2021
Accepted: 30 March 2021
Published: 1 April 2021

Publisher's Note: MDPI stays neutral with regard to jurisdictional claims in published maps and institutional affiliations.

Copyright: © 2021 by the authors. Licensee MDPI, Basel, Switzerland. This article is an open access article distributed under the terms and conditions of the Creative Commons Attribution (CC BY) license (http://creativecommons.org/licenses/by/4.0/).

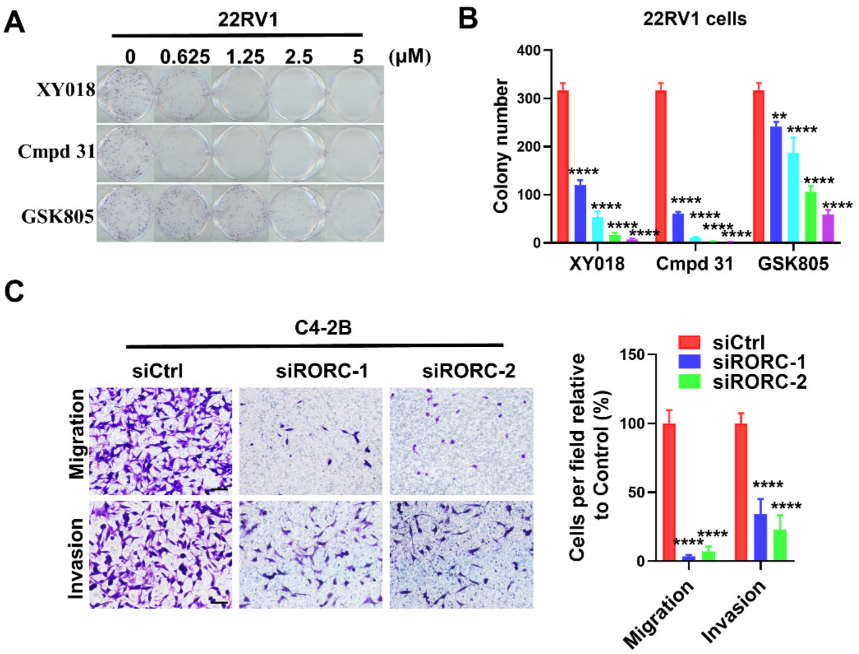

Figure S1. (**A**,**B**) 22Rv1 cells were seeded to 6 well plates at 500/well and treated with different doses of indicated compounds for 2 weeks. The colonies were then stained and counted. (**C**) C4-2B cells were transfected with control siRNA or two siRNAs targeting RORC for 48 h. Cells were later subjected to migration and invasion assay. After 24 h (for migration assay) or 48 h (for invasion assay), migrated or invasive cells were fixed and stained. Scale bar, 200 μm. Cells were counted under microscopy for five fields. Data were represented as mean ± S.D.** $p < 0.01$, *** $p < 0.001$.

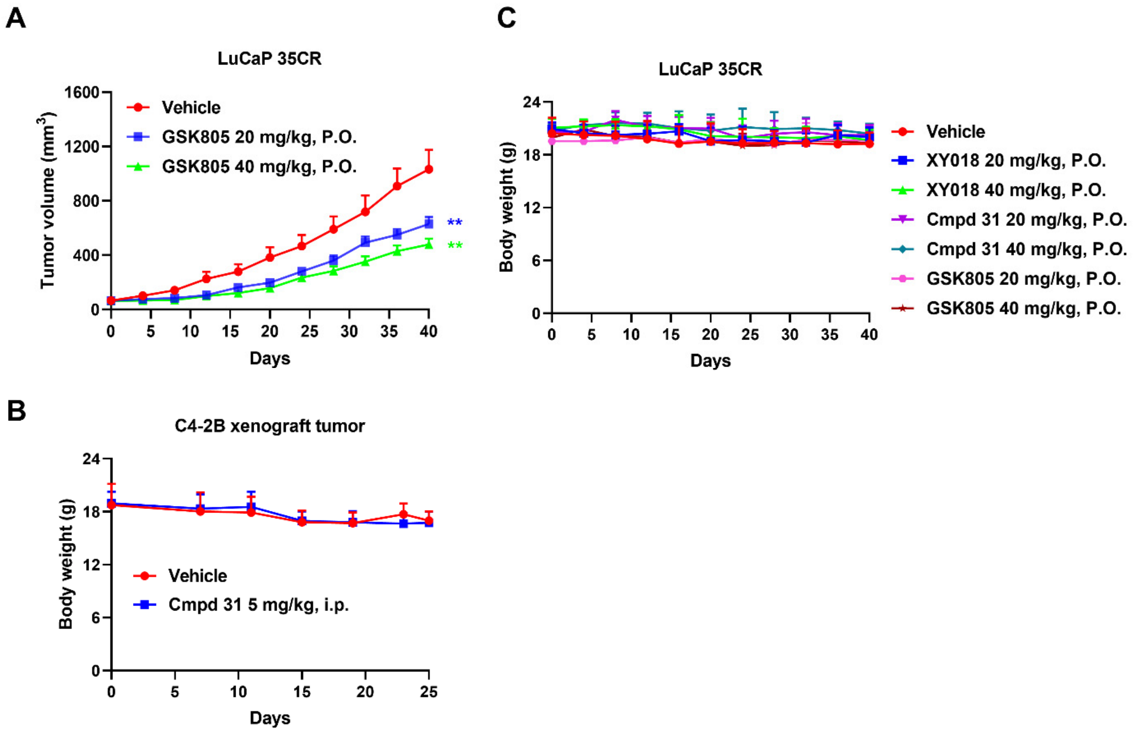

Figure S2. The effect of GSK805 on LuCaP 35CR tumors and the effect of RORγ antagonists on mouse bodyweight. (**A**) Mice with LuCaP-35CR PDX tumors were treated orally with three ROR-γ antagonists GSK805 (20 mg/kg or 40 mg/kg, five times per week) or vehicle (*n* = 8 tumors per group). Tumor volumes was monitored every 3 days. (**B**) Mice with C4-2B cell-derived tumors were treated with vehicle or 5 mg/kg Cmpd 31 (i.p.) five times per week for 25 days. Animal body weight was monitored. (**C**) Mice with LuCaP-35CR PDX tumors were treated orally with ROR-γ antagonists or vehicle as indicated. Animal body weight was monitored every 3 days. Results are presented as mean ± SD.

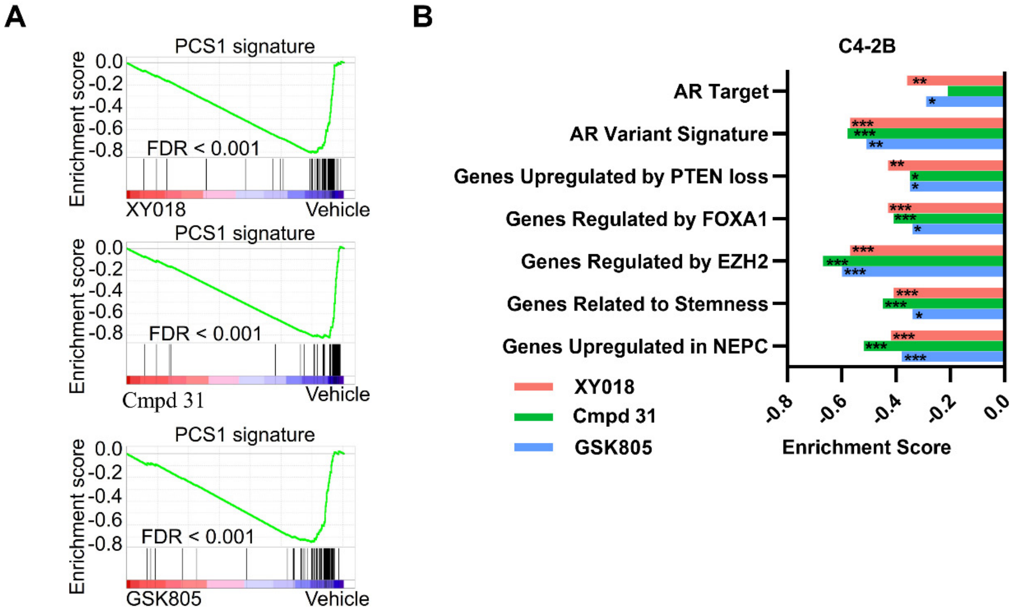

Figure S3. (**A**) GSEA of the PCS1 signature in C4-2B cells treated with 5 μM of XY018 (top), Cmpd 31 (middle) and GSK805 (bottom). (**B**) GSEA analysis of 7 sets of signature genes of PCa-relevant pathways, including the aggressive AR variant

and NEPC gene programs, in C4-2B cells treated with 5 μM of XY018, Cmpd 31 or GSK805, as compared to vehicle. * $p < 0.05$, ** $p < 0.01$, *** $p < 0.001$, **** $p < 0.0001$.

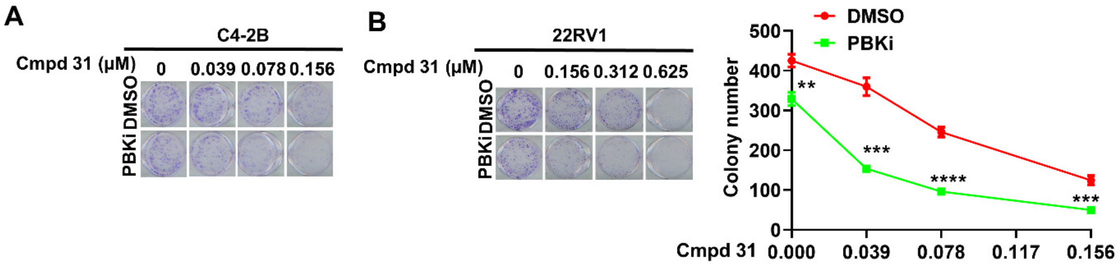

Figure S4. (**A**,**B**) C4-2B cells (**A**) and 22Rv1 (**B**) cells were seeded to 6 well plates at a density of 500 cells per well. After 24 h recovery, cells were incubated with indicated concentrations of PBKi and Cmpd 31 for 10 days. After stained with crystal violet, colonies were photoed and colonies number was counted.

Table S1. Antibodies used in immunoblotting

Antibody	Vendor	Catalogue Number	Dilution
GAPDH	Cell Signaling Technology	2118	1:4000
AR	Santa Cruz	Sc-7305	1:2000
ROR-γ	Ebioscience	14-6988-82	1:500
E-Cadherin	Santa Cruz	sc-8426	1:500
PTTG1	Cell Signaling Technology	13445S	1:1000
PBK	Cell Signaling Technology	4942T	1:1000
FN1	Santa Cruz	sc-69681	1:1000
NEK2	Santa Cruz	sc-55601	1:1000
MMP-1/8	Santa Cruz	sc-137044	1:500
MMP-2	Cell Signaling Technology	4022S	1:1000
MMP-3/10	Santa Cruz	sc-374029	1:500
MMP-11	Santa Cruz	sc-517445	1:500
MMP-12	Santa Cruz	sc-390863	1:500
c-Myc	Cell Signaling Technology	5605	1:1000
PARP	Cell Signaling Technology	9542	1:1000

Table S2. Primers for qRT-PCR

Gene	Forward Primer	Reverse Primer
STMN1	AAGGATCTTTCCCTGGAGGA	TGTGCCTCTCGGTTCTCTTT
MCM4	GGCTCTCATCGAGGCTTATG	TTCCACATCAATGGCTTCAA
CCNB1	CTTGCAGTAAATGATGTGGATG	GTGACTTCCCGACCCAGTAG
CDC6	TCTGATTCCCAAGAGGGTTG	CTGCCTGATCAAGAGCATCA
CDKN3	CATAGCCAGCTGCTGTGAAA	CCCGGATCCTCTTAGGTCTC
EZH2	AGGACGGCTCCTCTAACCAT	CTTGGTGTTGCACTGTGCTT
TPX2	TGGAAATATGCCCTTTCTCG	GCTTCCAAGTCTGTGCCTTC
FOXM1	TTCTCCTTTGCTTCCAGTTCA	CACTTTGATGGGTCTCGCTAA
KIF11	GAGGATTGGCTGACAAGAGC	GTTTGGCCATACGCAAAGAT
HMMR	TGGAAGCAAGGCTAAATGCT	ACCTGCAGCTTCATCTCCAT
MKI67	AAGCCCTCCAGCTCCTAGTC	TCCGAAGCACCACTTCTTCT
KNTC1	TGCAGCTCAAAGTCCACATC	GTCCATTTCCAGGTGCTGTT
RAB3B	CTTCCGCTATGCTGATGACA	GTAGCCCAGTCTTGGACAGC
SLC4A4	AGTCATCGTCCTTCCACCTG	GTTCGCTGCAATTCTTCACA

ANK3	GAATTCTGAGCAGCACCACA	TGGAATTTCCGTCTTCTTGG
GJB1	TCCCTGCAGCTCATCCTAGT	CCCTGAGATGTGGACCTTGT
SLC12A2	TGGTGGTGCAATTGGTCTAA	TTTTGCTTCCCACTCCATTC
CFD	GGAGCAGTGGGTGCTGAG	AGCTGTAGCAGCAGGAGGTC
COL6A1	CTGGGCGTCAAAGTCTTCTC	ATTCGAAGGAGCAGCACACT
PTGDS	AACCAGTGTGAGACCCGAAC	AGGCGGTGAATTTCTCCTTT
LTBP4	GAGGACGGCTACTCAGATGC	TTCGCAGGACCCATTAACTC
SOCS3	GCCACCTACTGAACCCTCCT	ACGGTCTTCCGACAGAGATG
SPEG	GTGGTCTCCTGGCTGAGAAA	CACTGCCGAGCACCATACT
GABRP	GGAGTTCACCTGGCTGAGAG	TCCTCCGAAGCTCAAACTGT
PENK	GCTGTCCAAACCAGAGCTTC	CTTCTGGCTCCATGGATAA
SMARCD3	CTCTGAAGAGGCCCATGAAG	GAACTTCCGCTTCTGTTTGC
CLIP3	AGGTTGGAGACCAGGTCCTT	TCCGCCAATCCTCTGAATAC
ACTC1	GCCCTGGATTTTGAGAATGA	ATGCCAGCAGATTCCATACC
ASPA	AACCCCAGAGCAGTGAAGAA	AGTGCACCCCATGTTAGAGG
COL4A6	TCTTTCCTCATGCACACTGC	TTCAGCGTTTCAGACACAGG
CYP4B1	CCTGGACAAAGTGGTGTCCT	CCAATCCACTGGAGGAAGAA
ROR2	GCCGCTACCATCAGTGCTAT	TCGGGGACGTTTTTATTCTG
SGCA	ACGTTTCTGAGCCTTCCTGA	TGTGACCTCAATGACCTGGA
SLC2A5	GCAACAGGATCAGAGCATGA	TGGAAACATGGACACGGTTA
PAGE4	TCCAGAGGAAGAGGAGATGG	CGCTCACTCCGAGTCTTTTC
ACOX2	ACTCCGCAGGAAAGTTGAGA	CTGAGGGCTCTCACGAAGAC
C16orf45	CCATGATGCCAGAGGAGATT	AGTTCTGCAGCTCCTGCTTC
GAPDH	GAAATCCCATCACCATCTTCC	ATGAGTCCTTCCACGATACCA
PTTG1	GGACCCCTCAAACAAAAACA	GAGAGGCACTCCACTCAAGG
PBK	TCTCATTCTCCTTGGGCTGT	AAAGGATCTTGGCTGGCTTT
FN1	CAGTGGGAGACCTCGAGAAG	GTCCCTCGGAACATCAGAAA
NEK2	TTGACCGGACCAATACAACA	CAGGAAAACATTGGCTGGTT
AR	ACATCAAGGAACTCGATCGTATCATTGC	TTG GGC ACT TGC ACA GAG AT
ARv7	CCATCTTGTCGTCTTCG-<br>GAAATGTTATGAAGC	TTT-<br>GAATGAGGCAAGTCAGCCTTTCT
RORC	GTGGGGACAAGTCGTCTGG	AGTGCTGGCATCGGTTTCG
KLK2	TCCAGATGTTGGGAGTAGGG	CGAACCAGAGGAGTTCTTGC
KLK3	TTGTCTTCCTCACCCTGTCC	TCACGCTTTTGTTCCTGATG
Clorf116	GCAGCTACGACTTCCTGTCC	TTCGTCCTTGCTGAGTGATG
PLK1	AAGAGATCCCGGAGGTCCTA	GCTGCGGTGAATGGATATTT
BIRC5	GGACCACCGCATCTCTACAT	TCTCCGCAGTTTCCTCAAAT
BUB1	CCTTTGGAGAACGCTCTGTC	GGAAGCTTGTGGAATGGTGT
CENPF	AAAGAAACAGACGGAACAACTG	CCAAGCAAAGACCGAGAACT
CENPA	GCACATCCTTTGGGAAGAGA	TCCGAAAGCTTCAGAAGAGC
CDC45	GCAGGTGAAGCAGAAGTTCC	AAGACATGGTGGCAAAGACC

Table S3. IC50 of RORγ antagonists in inhibition of C4-2B cell growth

	XY018	Cmpd 31	GSK805
IC_{50} (μM)	3.80 ± 0.35	1.51 ± 0.51	6.34 ± 0.68
